# Association of informal caregiving with body mass index and frequency of sporting activities: evidence of a population-based study in Germany

**DOI:** 10.1186/s12889-017-4786-6

**Published:** 2017-09-29

**Authors:** André Hajek, Jens-Oliver Bock, Hans-Helmut König

**Affiliations:** Department of Health Economics and Health Services Research, Hamburg Center for Health Economics, University Medical Center Hamburg-Eppendorf, Hamburg, Germany

**Keywords:** Informal care, Sports, Body-mass-index, Observational study

## Abstract

**Background:**

While most studies focused solely on the comparison between informal caregivers and non-caregivers, little is known about the relation between caregiving time or caregiving activities and lifestyle factors. Thus, the aim of this study was to examine whether informal caregiving time and type of caregiving activities are associated with body mass index (BMI) and the frequency of sporting activities among informal caregivers.

**Methods:**

Cross-sectional data were gathered from the German Ageing Survey, a nationally representative study among community-dwelling individuals aged ≥40 that includes a total of *n* = 1380 people who provide informal care services. Self-reported BMI and self-reported frequency of sporting activities (daily; several times a week; once a week; 1-3 times a month; less often; never) were used as dependent variables. The average time of providing informal care per week as well as four different caregiving activities (help around the house; looking after someone; performing nursing care services; help in another way) were included as independent variables. Multiple ordinal and linear regressions were used to estimate the association between caregiving factors and the frequency of sporting activities and BMI, respectively.

**Results:**

Among the 1380 informal caregivers, 65% provided help around the house, 83% looked after people, 28% provided nursing care services, and 68% provided any other help. Bivariate analyses showed that sporting activities and BMI differed by status of providing nursing care services, whereas the other three types of informal caregiving were not associated with BMI nor frequency of sporting activities except for the latter and provision of help around the house. Multiple regressions showed that BMI increased with caregiving time and performing nursing care services, whereas it was not associated with the other three caregiving activities. Likewise, the frequency of sporting activities decreased only with caregiving time and performing nursing care services.

**Conclusions:**

The present study revealed that caregiving time and performing nursing care services are associated with a higher BMI and a decreased frequency of sporting activities. As both, a higher BMI and fewer sporting activities are in turn related to various adverse health outcomes, this knowledge should be taken into account when planning informal caregiving.

**Electronic supplementary material:**

The online version of this article (10.1186/s12889-017-4786-6) contains supplementary material, which is available to authorized users.

## Background

Maintaining familiar surroundings or keeping social ties are main reasons why most people in need for care prefer to live at home as long as possible [1] where most of the required care is provided informally by family members [2]. Due to demographic aging, it is projected that the need for informal care will rise considerably in the upcoming decades, underlining the relevance of informal caregiving. Nevertheless, various studies have found that informal caregiving is associated with adverse outcomes, such as reduced satisfaction with life or mental health [3–5]. However, little is known about the relationship between informal caregiving and lifestyle factors such as body mass index (BMI) or frequency of sporting activities. An increased BMI is, for example, a risk factor for various cardiovascular diseases [6]. Sporting activities are protective against various adverse health outcomes [7]. Yet, this relationship might be worth investigating, since providing informal care limits the time disposable and motivation for doing sports.

For example, using data from the Behavioral Risk Factor Surveillance System (BRFSS), which is a nationally representative survey of the civilian, non-institutionalized, adult population (≥ 18 years) in the USA, Do et al. [8] found individuals who provided informal care to have a slightly higher BMI than those who do not. Also based on a US sample, Kusano et al. [9] showed that informal caregivers who suffered from a financial burden were more likely to be overweight and obese than the non-caregiving comparison group. Equally, Lee et al. [10] reported higher BMI rates for people who care for their spouses or parents as compared to a control group who do not. Moreover, Hoffman et al. [11] investigated sedentary behavior of caregivers and non-caregivers. Concerning sedentary behavior, they found that the percentages were similar in both groups.

While most studies focused solely on the comparison between informal caregivers and non-caregivers, little is known about the relation between caregiving time or caregiving activities and lifestyle factors. This knowledge is important to reveal further insights into the relation between conditions of informal care and these outcome measures. Hence, the aim of the present study was to determine whether caregiving time and caregiving activities are associated with two outcome measures ((1) BMI and the (2) frequency of sporting activities) among informal caregivers using a representative sample of community-dwelling individuals in the second half of life (aged 40 and over) in Germany. Thus, our aim was twofold: First, to study the association between caregiving time as well as caregiving activities and BMI. Second, to study the association between caregiving as well as caregiving activities and frequency of sporting activities.

It is important to know whether informal caregiving time is associated with, e.g., restrictions in the frequency of sporting activities and an increased BMI because this might help to identify caregivers at risk for an unhealthy lifestyle. This unhealthy lifestyle is in turn associated with morbidity and mortality [12, 13]. In addition, it is important to know which caregiving activities are associated with BMI as well as the frequency of sporting activities as this may reveal which caregiving tasks pose a particular challenge for individuals providing informal care.

## Methods

### Sample

Cross-sectional data from the fifth wave of the German Ageing Survey (Deutscher Alterssurvey; DEAS) were used. DEAS is an ongoing longitudinal, population-based study of the German community-dwelling population in the second half of life (40 years and over) started in 1996. The sample was drawn by means of national probability sampling and was systematically stratified by region (West and East Germany), age and gender. Follow-up waves took place in 2002 (second wave), 2008 (third wave), 2011 (fourth wave), and 2014 (fifth wave). Each wave comprised a panel-sample (participants who had already been interviewed) and a new cross-sectional sample, except for 2011, which was a pure panel survey. Face-to-face interviews are conducted in each wave. After the oral interview, respondents are asked to fill out an additional written questionnaire (so called ‘drop-off’ self-report questionnaire). Please see for further details Klaus et al. [14].

In the fifth wave, about 6000 participants were interviewed for the first time (response rate: 25%) while over 4000 participants had already been interviewed in former waves (response rate: 61%). In total, the response rate corresponds to that in other large German survey studies [15]. Out of the 10,324 participants in the fifth wave, the subsample of *n* = 1380 who provided informal care and filled out the ‘drop-off’ self-report questionnaire including, e.g., psychological scales and physical (chronic) illnesses, was used for the present analysis.

Please note that an ethical statement for the DEAS study was not necessary because criteria for the need of an ethical statement were not met (risk for the respondents, lack of information about the aims of the study, examination of patients).

### Dependent variables: Body-mass-index and frequency of sporting activities

The BMI was computed from self-reported height (meter) and weight (kg) as weight divided by height-squared. In adults, the BMI is widely used as a body weight classification system (e.g., for excess weight). Furthermore, individuals were asked “How often do you do sports such as hiking, soccer, gymnastics, or swimming?” (daily; several times a week; once a week; 1-3 times a month; less often; never). The frequency format is based on the International Physical Activity Questionnaire (IPAQ) [16]. Furthermore, the item used in our study was validated [17].

In additional analysis, the frequency of sporting activities was replaced by three different outcomes; including frequency of … (1) strenuous physical activities, (2) moderate physical activities, and (3) light physical activities. Again, the frequency format was: daily; several times a week; once a week; 1-3 times a month; less often; never.

The exact wording was as follows:

- “How often do you do strenuous physical activities? What is meant here are activities that require great physical efforts and whereby you breathe more heavily than normal, e.g. carrying heavy loads, arduous gardening, aerobic or fast cycling. Please include all strenuous physical activities, those within your work, in the household and in the garden, in order to get from one place to another and in your spare time.”
- “How often do you do moderate physical activities? What is meant here are activities that require moderate physical efforts whereby you breathe a bit more heavily than normal, e.g. carrying light loads, easy gardening, hiking, swimming or cycling with normal speed. Please include all moderate physical activities, those within your work, in the household and in the garden, in order to get from one place to another and in your spare time. Please don’t include walks.”
- “How often do you do light physical activities? What is meant here are activities that require little physical efforts whereby you don’t breathe more heavily than normal, e.g. walking around at home, walks from one place to another as well as walks that you do for relaxation, as sport, as training or just for pleasure.”

### Main independent variables: Informal caregiving activities and caregiving time

Individuals were asked if they provide informal care with the question: “Are there people you look after or care for regularly due to their poor state of health, either on a private or volunteer basis?” (no; yes). Among those informal caregivers, individuals were asked (yes; no) “What kind of care or assistance do you provide to the person who you help? A: Do you help around the house? B: Do you look after him/her or keep him/her company? C: Do you perform care services for the person you assist? D: Do you help in another way?” In a narrow sense, care can be seen as “nursing care” (C: “Do you perform care services for the person you assist?”).

Furthermore, informal caregivers should rate how much time they spend per week providing informal care (average number of hours per week, ranging from 0 to 168 h).

It is worth noting that the respondents were also asked ‘Who do you assist in this way?’ (Possible to name up to three persons). However, which is also worth emphasizing, our main independent variables (informal caregiving activities and caregiving time) refer to the person who the caregiver help *the most*.

### Independent variables: Potential confounders

Based on theoretical considerations and previous findings the following socioeconomic variables were included in the regression analysis: Age, sex, family status (distinguishing between: married and living together with spouse; others (married and living separately, divorced, widowed, and single)), occupational status (employed, retired, other), and individual monthly net equivalent income (OECD scale). Moreover, the sum of physical illnesses (e.g., insomnia, eye problems, ear problems, cardiac and circulatory disorders or cancer; ranging from 0 to 11) was used as covariate. For example, it has been shown that age is associated with physical activities [18]. Furthermore, it has been shown that BMI is positively associated with younger age [19]. Moreover, it has been found that employment status is associated with physical activity [20]. In addition, it has been demonstrated that (1) cardiac and circulatory disorders, (2) respiratory problems, asthma, shortness of breath, (3) stomach and intestinal problems, (4) cancer, and (5) gall bladder, liver or kidney problems are associated with weight loss [21, 22].

### Statistical analysis

First, descriptive sample statistics were computed. Second, multiple ordinal (ordered probit, with frequency of sports activities as outcome variable) and linear regressions (with BMI as outcome variable) were estimated, adjusting for potential confounders. The statistical significance was determined with *p* < 0.05. Stata 14.0 (StataCorp, College Station, Texas, USA) was used to conduct statistical analyses.

## Results

### Sample characteristics

Table 1 shows the sample characteristics stratified by type of informal caregiving. Among the caregivers, 65.1% provided help around the house, 83.2% looked after their relative, 27.9% provided nursing care services, and 67.9% provided any other type of help. The mean age of all informal caregivers was 63.4 years (SD: 10.4 years) with a range from 40 to 91 years. 60.0% were female.

**Table 1:** Sample characteristics by type of informal caregiving

	Help around house (*N* = 1378)					Looking after someone (*N* = 1379)					Nursing care services (*N* = 1380)					Any other help (*N* = 1378)					Missings
	yes (*n* = 897)		no (*n* = 481)		*p*-value	yes (*n* = 1147)		no (*n* = 232)		*p*-value	yes (*n* = 385)		no (*n* = 995)		*p*-value	yes (*n* = 935)		no (*n* = 443)		*p*-value	
	N/Mean	%/SD	N/Mean	%/SD	t-test/chi^2^	N/Mean	%/SD	N/Mean	%/SD	t-test/chi^2^	N/Mean	%/SD	N/Mean	%/SD	t-test/chi^2^	N/Mean	%/SD	N/Mean	%/SD	t-test/chi^2^	
Sex: female	557	62.1%	272	56.6%	.045	702	61.2%	128	55.2%	.087	264	68.6%	565	56.8%	<.001	565	30.4%	263	59.4%	.707	0.0%
Age in years	62.5	10.5	65.1	9.9	<.001	63.4	10.2	63.5	11.0	.878	64.7	10.7	63.0	10.2	.006	63.1	10.2	64.1	10.6	.098	0.0%
Marital status:																					
- married and living together with spouse	678	75.6%	346	72.2%	.225	856	77.8%	168	72.4%	.421	304	78.9%	721	72.6%	.045	713	76.4%	311	70.2%	.180	0.1%
- married and not living together with spouse	19	2.1%	11	2.3%		27	2.4%	3	1.3%		11	2.9%	19	1.9%		19	2.0%	11	2.5%		
- divorced	75	8.4%	45	9.4%		101	8.8%	20	8.6%		23	5.9%	97	9.7%		74	7.9%	47	10.6%		
- widowed	59	6.6%	47	9.8%		81	7.1%	24	10.3%		26	6.8%	80	8.1%		66	7.1%	39	8.8%		
- single	66	7.4%	30	6.3%		80	6.9%	17	7.3%		21	5.5%	76	7.7%		61	6.5%	35	7.9%		
Monthly net equivalent income in Euro	1947.9	1406.1	2226.8	1517.7	<.001	2068.5	1534.5	1934.8	929.9	.214	1832.8	1364.9	2128.9	1475.0	<.001	2017.8	1366.0	2101.8	1613.6	.329	5.6%
Number of physical illnesses	2.7	1.9	2.4	1.7	.002	2.6	1.8	2.5	1.9	.904	2.7	1.9	2.5	1.8	.042	2.6	1.8	1.4	1.8	.109	1.9%
Occupational status:																					
- employed	384	42.9%	161	33.5%	<.001	455	39.6%	91	39.4%	.245	125	32.5%	420	42.3%	.044	378	40.5%	167	37.7%	.306	0.3%
- retired	408	45.5%	276	57.4%		562	49%	122	52.8%		212	55.1%	474	47.7%		451	48.3%	233	52.6%		
- other	104	11.6%	44	9.2%		130	11.3%	18	7.8%		48	12.5%	100	10.15		105	11.2%	43	9.7%		
Body mass index	26.97	4.59	26.49	4.28	.061	26.8	4.6	26.7	4.2	.623	27.3	4.6	26.6	4.4	.022	26.8	4.6	26.7	4.3	.620	1.7%
Frequency of sports activities:																					
- everyday	73	8.1%	52	10.8%	.001	103	9.0%	22	9.5%	.721	30	7.8%	95	9.6%	<.001	86	9,2%	39	8.8%	.791	0.0%
- several times a week	237	26.4%	158	32.9%		327	28.5%	70	30.2%		86	22.3%	310	31.2%		260	27.8%	136	30.7%		
- once a week	175	19.5%	92	19.1%		216	18.8%	51	22.0%		75	19.5%	192	19.3%		180	19.3%	88	19.9%		
- 1 to 3 times a month	77	8.6%	38	7.9%		97	8.5%	18	7.8%		25	6.5%	90	9.1%		83	8.9%	31	7.0%		
- less common	101	11.3%	61	12.7%		140	12.2%	22	9.5%		38	10.0%	124	12.5%		111	11.9%	51	11.5%		
- never	237	26.1%	80	16.6%		264	23.0%	49	21.1%		131	34.0%	184	18.5%		215	23.0%	98	22.1%		

The BMI was 26.8 kg/m^2^ (SD: 4.5 kg/m^2^) on average. As Table 1 displays, there was a statistical significant difference between caregivers who provide nursing care services as compared to those who only provide other types of informal care; the BMI of the group providing nursing care services was about 0.7 kg/m^2^ higher than the BMI of the group not providing nursing care services. Equally, there was a significant deviation in the frequency of doing sports between both aforementioned groups. Additionally, the frequency of doing sports deviated between the groups who provided help around the house and those who did not. With respect to other types of informal caregiving, there were no significant differences regarding frequency of doing sporting activities and BMI.

Table 2 splits the sample by the median of informal caregiving time per week. The median was 5 h per week and people who provided informal care of exactly 5 h per week were assigned to the group below the median so that this group is larger with *n* = 732 participants compared to *n* = 604 participants above the median. The latter group above the median was more likely to be female and somewhat older. In addition, there were significant differences in all considered potentially confounding variables. The BMI was significantly higher in the group that provided care more than 5 h per week. Besides, the frequency of doing sports deviated significantly between the two groups.

**Table 2:** Sample characteristics by the time spent on informal caregiving per week (median split)

	Time spent on informal caregiving per week (*N* = 1336)					
	≤5 h (*n* = 732)		> 5 h (*n* = 604)		*p*-value	Missings
	N/Mean	%/SD	N/Mean	%/SD	t-test/chi^2^	
Sex: female	422	57.7%	382	63.3%	.038	0.0%
Age in years	62.1	10.2	64.5	10.3	<.001	0.0%
Marital status:						
- married and living together with spouse	519	71.0%	473	78.4%	.001	0.1%
- married and not living together with spouse	11	1.5%	18	2.9%		
- divorced	81	11.1%	37	6.1%		
- widowed	66	9.0%	37	6.1%		
- single	54	7.4%	38	6.3%		
Monthly net equivalent income in Euro	2179.1	1494.6	1899.8	1420.6	<.001	5.6%
Number of physical illnesses	2.3	1.7	2.8	1.9	<.001	1.9%
Occupational status:						
- employed	349	47.7%	193	31.9%	<.001	0.3%
- retired	321	43.9%	329	54.5%		
- other	61	8.3%	82	13.6%		
Body mass index	26.5	4.2	27.3	4.7	.001	1.7%
Sport: - daily	73	10.0%	48	7.9%	<.001	0.0%
- more than once a week	257	35.1%	128	21.2%		
- once a week	140	19.1%	123	20.4%		
- 1-3 times a month	65	8.9%	49	8.1%		
- less than 1-3 times a month	83	11.3%	72	11.9%		
- never	114	15.6%	184	30.5%		

### Multiple regression analysis

Table 3 displays the results of the multiple regression analyses with the BMI as dependent variable. Model 1 to 4 (marked as (1), (2), (3), and (4) in Table 3)) include the types of informal caregiving, model 5 (marked as (5) in Table 3) the time of informal caregiving per week. The same principle holds for Table 4. All models are fully adjusted for the various potential confounders (sex, age, marital status, number of illnesses, income, and occupational status). The regression models showed no association of BMI with three out of the four types of informal caregiving. Only the provision of nursing care services was associated with an increased BMI of about 0.6 kg/m^2^. For example, for an individual of 1.70 m height performing nursing care services this increase in BMI equals an increase in weight of about 1.68 kg (ceteris paribus). Spending more time on providing informal care was associated with an increased BMI; each additional hour per week was associated with a higher BMI of about 0.01 kg/m^2^.

**Table 3:** Multiple linear regression analyses with body mass index as dependent variable

	(1)	(2)	(3)	(4)	(5)
Independent variables	Dependent variable: body mass index (BMI)				
Sex: female (Ref.: male)	-0.908***	-0.919***	-0.967***	-0.925***	-1.001***
	(0.251)	(0.252)	(0.253)	(0.250)	(0.255)
Age in years	-0.043*	-0.043*	-0.045*	-0.044*	-0.039+
	(0.021)	(0.021)	(0.021)	(0.021)	(0.021)
Marital status: - married, not living together with spouse (Ref.: married and living together with spouse)	-0.518	-0.526	-0.563	-0.502	-0.562
	(0.774)	(0.774)	(0.781)	(0.775)	(0.780)
- divorced	-0.764	-0.832+	-0.697	-0.823+	-0.690
	(0.472)	(0.471)	(0.472)	(0.471)	(0.476)
- widowed	0.373	0.407	0.419	0.278	0.384
	(0.481)	(0.485)	(0.481)	(0.477)	(0.482)
- single	-0.128	-0.155	-0.130	-0.294	0.0773
	(0.608)	(0.602)	(0.602)	(0.597)	(0.622)
Number of illnesses	0.461***	0.470***	0.459***	0.473***	0.455***
	(0.078)	(0.077)	(0.077)	(0.076)	(0.078)
Mean monthly net equivalent income	-0.359***	-0.366***	-0.350***	-0.370***	-0.378***
	(0.088)	(0.089)	(0.085)	(0.090)	(0.079)
Occupational status: - retired (Ref.: employed)	0.148	0.125	0.102	0.128	0.0376
	(0.440)	(0.439)	(0.436)	(0.437)	(0.437)
- others	0.322	0.316	0.300	0.359	0.184
	(0.506)	(0.506)	(0.504)	(0.505)	(0.506)
Help around house: yes (Ref.: no)	0.132				
	(0.259)				
Looking after someone: yes (Ref.: no)		0.285			
		(0.319)			
Nursing care services: yes (Ref.: no)			0.580*		
			(0.294)		
Any other help: yes (Ref.: no)				0.020	
				(0.255)	
Time per week spent for informal care (in hours)					0.013*
					(0.006)
Constant	29.52***	29.36***	29.59***	29.61***	29.37***
	(1.173)	(1.163)	(1.157)	(1.190)	(1.181)
Observations	1,256	1,257	1,257	1,256	1,221
R²	0.064	0.065	0.066	0.066	0.068

Comments: Beta-coefficients were reported. Cluster-robust standard errors in parentheses

***p < 0.001

**p < 0.01

*p < 0.05, + *p* < 0.10

**Table 4:** Multiple ordered probit regression analyses with frequency of sports activities per week as dependent variable

	(1)	(2)	(3)	(4)	(5)
Independent variables	Dependent variable: Frequency of sports activities				
Sex: female (Ref.: male)	0.121+	0.110+	0.149*	0.110+	0.166**
	(0.063)	(0.063)	(0.063)	(0.063)	(0.064)
Age in years	−0.012*	−0.011*	−0.010*	−0.011*	−0.008
	(0.005)	(0.005)	(0.005)	(0.005)	(0.005)
Marital status: - married, not living together with spouse (Ref.: married and living together with spouse)	0.0756	0.0778	0.0956	0.0716	0.204
	(0.203)	(0.203)	(0.204)	(0.203)	(0.102)
- divorced	0.139	0.159	0.122	0.156	0.204
	(0.107)	(0.107)	(0.107)	(0.107)	(0.073)
- widowed	−0.119	−0.0895	−0.123	−0.0830	0.108
	(0.116)	(0.116)	(0.116)	(0.116)	(−0.179)
- single	−0.216+	−0.205+	−0.219+	−0.214+	0.118
	(0.117)	(0.117)	(0.117)	(0.117)	(−0.217)
Number of illnesses	−0.050**	−0.055**	−0.055**	−0.056**	−0.048**
	(0.018)	(0.017)	(0.017)	(0.017)	(0.018)
Mean monthly net equivalent income	0.068**	0.075***	0.068**	0.074***	0.076***
	(0.021)	(0.021)	(0.021)	(0.021)	(0.021)
Occupational status: - retired (Ref.: employed)	0.154	0.169+	0.181+	0.173+	0.208
	(0.101)	(0.101)	(0.101)	(0.101)	(0.102)
- others	−0.113	−0.113	−0.102	−0.113	−0.065
	(0.107)	(0.107)	(0.107)	(0.107)	(0.109)
Help around house: yes (Ref.: no)	−0.225***				
	(0.063)				
Looking after someone: yes (Ref.: no)		−0.062			
		(0.080)			
Nursing care services: yes (Ref.: no)			−0.284***		
			(0.068)		
Any other help: yes (Ref.: no)				−0.024	
				(0.064)	
Time per week spent for informal care (in hours)					−0.011***
					(0.002)
Constant cut1	−1.511***	−1.333***	−1.312***	−1.313***	−1.189***
	(0.281)	(0.280)	(0.275)	(0.279)	(0.280)
Constant cut2	−1.141***	−0.966***	−0.941***	−0.945***	−0.814**
	(0.280)	(0.278)	(0.273)	(0.278)	(0.279)
Constant cut3	−0.913**	−0.739**	−0.713**	−0.720**	−0.577*
	(0.280)	(0.278)	(0.273)	(0.278)	(0.279)
Constant cut4	−0.416	−0.245	−0.217	−0.224	−0.066
	(0.279)	(0.278)	(0.273)	(0.277)	(0.279)
Constant cut5	0.617*	0.790**	0.820**	0.809**	0.982***
	(0.281)	(0.280)	(0.275)	(0.279)	(0.281)
Observations	1273	1274	1275	1273	1236
Pseudo R^2^	0.014	0.011	0.016	0.012	0.021

Comments: Coefficients were reported (larger values correspond to “higher” outcomes). 95% confidence intervals in parentheses

***p < 0.001

**p < 0.01

*p < 0.05, + *p* < 0.10

With respect to the control variables, female gender, the number of illnesses, and net equivalent income per capita were positively associated with the BMI, whereas a higher age was negatively associated with the BMI in all five models.

The results of multiple ordered probit regression analyses with frequency of doing sporting activities are depicted in Table 4. Help around the house was associated with a lower frequency of doing sports activities (model (1)) after adjustment of the covariates. Equally, providing nursing care services was associated with doing fewer sporting activities (model (3)). The informal caregiving types of ‘looking after someone’ and ‘any other help’ were not associated with the frequency of doing sporting activities (models (2) and (4)). With respect to the time spent on informal caregiving, each additional hour spent on informal caregiving was associated with a lower frequency of sporting activities (model (5)).

Regarding the control variables, a higher number of illness was associated with a lower frequency of doing sports in all five models, and higher net equivalent income per capita was associated with higher frequency.

In additional analysis (please see Additional file 1: Tables S1, Additional file 2: Tables S2 and Additional file 3: Tables S3), the frequency of sporting activities was replaced by the frequency of (1) light (2) moderate, and (3) strenuous physical activities. While providing nursing care services and caregiving time were associated with the frequency of light physical activities, only caregiving time was significantly associated with the frequency of moderate physical activities. In contrast, none of the caregiving factors was associated with the frequency of strenuous physical activities.

## Discussion

### Main findings

Using a representative sample of individuals in the second half of life, this study aimed at examining whether caregiving time and caregiving activities are associated with BMI and the frequency of sporting activities. The results show that the BMI increased with caregiving time and performing nursing care services, whereas it was not associated with the other caregiving activities (help around house; looking after someone; any other help). Equally, the frequency of sporting activities decreased with caregiving time and performing nursing care services, whereas the outcome measure was not associated with the other caregiving activities (except for help around house). Additional analysis showed that nursing care services were only associated with the frequency of light physical activities, whereas it was neither significantly associated with moderate nor with strenuous physical activities.

### Previous research

While several studies examined whether informal caregivers and non-caregivers differ in lifestyle factors [11, 23], little is known about the associations between caregiving time as well as caregiving activities with lifestyle factors among informal caregivers. For example, it was found that 26.1% of full-time informal caregivers were obese compared with 22.6% of part-time informal caregivers in Thailand (*n* = 60,569) [24]. In addition, it has been shown that women providing care to an ill/disabled spouse had a higher BMI (mean BMI was 27.0) compared to women providing care to an ill parent/ill others (both, with mean BMI of 26.1) in 11 states of the USA (*n* = 54,411) [10]. However, physical activity (metabolic equivalent hours (MET-hours) per week) was similar between women providing care to an ill/disabled spouse and women providing care to an ill parent.

Our study adds further insights into the relationship between these lifestyle factors and informal caregiving time as well as several caregiving activities. The current study showed that the BMI is positively associated with caregiving time and performing nursing care services, whereas it was not associated with the other caregiving activities. A possible explanation might be that long caregiving time as well as performing nursing care services are associated with adverse factors such as stress or depressive symptoms [3] because (1) informal caregivers are generally not used to perform nursing caregiving activities and because (2) an increase in informal caregiving time is often the result of an increase in cognitive impairment of care-recipients [25]. This is supported by the fact that according to our calculations (German Ageing Survey, fifth wave), caregiving time was associated with increased depressive symptoms [26] (*r* = .19, *p* < .001) and increased stress [27] (*r* = .16, *p* < .001). These factors including depressive symptoms are in turn associated with a higher BMI [28] or a reduced frequency of physical activities [29].

Likewise, the frequency of sporting activities decreased with caregiving time and performing nursing care services, whereas it was not associated with the other caregiving activities. In the German Ageing Survey (fifth wave), the evaluation of leisure time (ranging from 1 = “very bad” to 5 = “very good”) was associated with caregiving time (*r* = −.30, *p* < .001). This might support the idea that an increase in caregiving time restricts leisure time. Furthermore, performing nursing care activities might be a source of stress, which might exhaust individuals’ resources. This is supported by the fact that performing nursing care services was associated with increased perceived stress (*r* = .13, *p* < .001) in the German Ageing Survey (fifth wave).

Thus, individuals might have a lack of energy or they might be too tired to exercise. However, this is speculative and consequently should be investigated in future studies. It should be acknowledged that caregiving time and performing nursing care services were only weakly associated with both outcome measures. For example, the partial η^2^ for these two explanatory variables was .003 each (0.3% of the variability in BMI explained) in the models presented in column 3 and column 5 of Table 3. Consequently, the clinical significance might be limited or even small.

In contrast to performing nursing care activities, the other caregiving activities (except for help around house) might have a stronger focus on supervision or are strongly related to everyday household activities. In many cases, informal caregivers might be familiar with these activities, which might help to cope with the demands of informal care. Thus, it is assumed that individuals did not perceive the other caregiving activities as demanding, stressful or challenging. Consequently, it appears plausible that the other caregiving activities are not associated with both outcome measures.

### Strengths and limitations

This is one of few studies providing insights into the relationship between informal caregiving time, caregiving activities and the lifestyle factors of BMI and sporting activities. Data from the present study were gathered from a large, population-based study among community-dwelling individuals aged 40 and over. Data on caregiving time as well as on four caregiving activities were provided. A limitation of the study is that we cannot exclude a potential recall bias in participants’ estimations of average caregiving time. Thus, it is also likely that self-reported BMI is biased downwards because subjects generally tend to underestimate weight and overestimate height [30]. In addition, this study is cross-sectional, which does not allow analyzing the causal inference of the variables of interest. Thus, we cannot dismiss the possibility that changes in body mass index (BMI) and changes in the frequency of sporting activities affect the probability of starting informal caregiving. However, we assume that this is rarely the case.

Moreover, because a sample selection bias cannot be ruled out, our findings might be difficult to generalize to individuals with, e.g., low education. However, it has been shown that selectivity effects were rather small in the DEAS study [31].

## Conclusion

The present study revealed that caregiving time and performing nursing care services are associated with a higher BMI and a decreased frequency of sporting activities. As both, a higher BMI and fewer sporting activities are in turn related to various adverse health outcomes (including morbidity and mortality), this knowledge should be taken into account by policy makers when planning and promoting informal caregiving. For example, the provision of short-term accommodation (respite care) might be fruitful in reducing symptoms of stress in informal caregivers [32]. Future research is required to disentangle the immediate impact of informal caregiving on physical activities and the long-term impact on BMI (which might be mediated by physical activities).

## Additional files

Additional file 1: Multiple ordered probit regression analyses with frequency of light physical activities as dependent variable. (DOCX 15 kb)

Additional file 2: Multiple ordered probit regression analyses with frequency of moderate physical activities as dependent variable. (DOCX 15 kb)

Additional file 3: Multiple ordered probit regression analyses with frequency of strenuous physical activities as dependent variable. (DOCX 15 kb)

## Abbreviations

BMI: body mass index
BRFSS: Behavioral Risk Factor Surveillance System
DEAS: German Ageing Survey
OECD: Organization for Economic Co-Operation and Development
