# Supplementary material for: Association of informal caregiving with body mass index and frequency of sporting activities: evidence of a population-based study in Germany
Source: BMC Public Health. 2017 Sep 29;17:755. doi: 10.1186/s12889-017-4786-6 (PMC5622456; doi:10.1186/s12889-017-4786-6)
Supplement: Supplementary file 1 — Multiple ordered probit regression analyses with frequency of light physical activities as dependent variable. (DOCX 15 kb) [file 12889_2017_4786_MOESM1_ESM.docx]

Additional file 1. Multiple ordered probit regression analyses with frequency of light physical activities as dependent variable

|  | (1) | (2) | (3) | (4) | (5) |
| --- | --- | --- | --- | --- | --- |
| Independent variables | Dependent variable: Frequency of light physical activities | | | | |
| Sex: female (Ref.: male) | 0.197** | 0.195** | 0.209** | 0.198** | 0.234** |
|  | (0.059 - 0.334) | (0.057 - 0.333) | (0.071 - 0.348) | (0.061 - 0.336) | (0.094 - 0.374) |
| Age in years | -0.003 | -0.003 | -0.003 | -0.003 | -0.002 |
|  | (-0.014 - 0.008) | (-0.014 - 0.007) | (-0.013 - 0.008) | (-0.014 - 0.007) | (-0.013 - 0.008) |
| Marital status: - married, not living together with spouse (Ref.: married and living together with spouse) | -0.179 | -0.181 | -0.174 | -0.188 | -0.165 |
|  | (-0.625 - 0.267) | (-0.627 - 0.265) | (-0.619 - 0.272) | (-0.634 - 0.258) | (-0.612 - 0.282) |
| - divorced | 0.056 | 0.066 | 0.041 | 0.057 | 0.026 |
|  | (-0.187 - 0.300) | (-0.177 - 0.308) | (-0.202 - 0.285) | (-0.186 - 0.300) | (-0.219 - 0.271) |
| - widowed | -0.087 | -0.093 | -0.099 | -0.092 | -0.135 |
|  | (-0.337 - 0.163) | (-0.344 - 0.157) | (-0.349 - 0.151) | (-0.343 - 0.158) | (-0.390 - 0.119) |
| - single | -0.159 | -0.148 | -0.155 | -0.118 | -0.146 |
|  | (-0.413 - 0.096) | (-0.401 - 0.105) | (-0.408 - 0.099) | (-0.374 - 0.138) | (-0.407 - 0.115) |
| Number of illnesses | -0.017 | -0.019 | -0.016 | -0.018 | -0.015 |
|  | (-0.055 - 0.022) | (-0.058 - 0.019) | (-0.054 - 0.022) | (-0.056 - 0.021) | (-0.054 - 0.024) |
| Mean monthly net equivalent income | 0.000 | 0.000 | 0.000 | 0.000 | 0.000 |
|  | (-0.000 - 0.000) | (-0.000 - 0.000) | (-0.000 - 0.000) | (-0.000 - 0.000) | (-0.000 - 0.000) |
| Occupational status: - retired (Ref.: employed) | 0.031 | 0.043 | 0.048 | 0.036 | 0.088 |
|  | (-0.194 - 0.256) | (-0.181 - 0.267) | (-0.176 - 0.273) | (-0.188 - 0.260) | (-0.139 - 0.315) |
| - others | 0.090 | 0.091 | 0.099 | 0.086 | 0.128 |
|  | (-0.154 - 0.335) | (-0.153 - 0.336) | (-0.146 - 0.343) | (-0.159 - 0.330) | (-0.121 - 0.378) |
| Help around house: yes (Ref.: no) | 0.039 |  |  |  |  |
|  | (-0.103 - 0.181) |  |  |  |  |
| Looking after someone: yes (Ref.: no) |  | -0.016 |  |  |  |
|  |  | (-0.190 - 0.158) |  |  |  |
| Nursing care services: yes (Ref.: no) |  |  | 0.155* |  |  |
|  |  |  | (0.007 - 0.303) |  |  |
| Any other help: yes (Ref.: no) |  |  |  | 0.077 |  |
|  |  |  |  | (-0.065 - 0.219) |  |
| Time per week spent for informal care (in hours) |  |  |  |  | -0.006** |
|  |  |  |  |  | (-0.009 - -0.002) |
| Constant cut1 | -2.146*** | -2.231*** | -1.907*** | -2.130*** | -2.179*** |
|  | (-2.795 - -1.496) | (-2.900 - -1.561) | (-2.596 - -1.218) | (-2.783 - -1.477) | (-2.822 - -1.536) |
| Constant cut2 | -1.502*** | -1.588*** | -1.259*** | -1.472*** | -1.533*** |
|  | (-2.136 - -0.869) | (-2.242 - -0.934) | (-1.934 - -0.583) | (-2.108 - -0.836) | (-2.159 - -0.906) |
| Constant cut3 | -1.253*** | -1.339*** | -1.008** | -1.221*** | -1.281*** |
|  | (-1.883 - -0.622) | (-1.990 - -0.687) | (-1.681 - -0.335) | (-1.854 - -0.587) | (-1.904 - -0.658) |
| Constant cut4 | -1.028** | -1.114*** | -0.782* | -0.994** | -1.047*** |
|  | (-1.657 - -0.398) | (-1.764 - -0.463) | (-1.454 - -0.109) | (-1.626 - -0.362) | (-1.668 - -0.425) |
| Constant cut5 | -0.300 | -0.386 | -0.053 | -0.265 | -0.324 |
|  | (-0.928 - 0.329) | (-1.035 - 0.263) | (-0.725 - 0.619) | (-0.896 - 0.366) | (-0.945 - 0.297) |
|  |  |  |  |  |  |
| Observations | 1,236 | 1,237 | 1,238 | 1,236 | 1,200 |
| Pseudo R² | 0.005 | 0.005 | 0.007 | 0.006 | 0.010 |

Comments: Coefficients were reported (larger values correspond to "higher" outcomes). 95% confidence intervals in parentheses. *** p<0.001, ** p<0.01, * p<0.05, + p<0.10.
