# Supplementary material for: Association of informal caregiving with body mass index and frequency of sporting activities: evidence of a population-based study in Germany
Source: BMC Public Health. 2017 Sep 29;17:755. doi: 10.1186/s12889-017-4786-6 (PMC5622456; doi:10.1186/s12889-017-4786-6)
Supplement: Supplementary file 2 — Multiple ordered probit regression analyses with frequency of moderate physical activities as dependent variable. (DOCX 15 kb) [file 12889_2017_4786_MOESM2_ESM.docx]

Additional file 2. Multiple ordered probit regression analyses with frequency of moderate physical activities as dependent variable

|  | (1) | (2) | (3) | (4) | (5) |
| --- | --- | --- | --- | --- | --- |
| Independent variables | Dependent variable: Frequency of moderate physical activities | | | | |
| Sex: female (Ref.: male) | 0.087 | 0.079 | 0.088 | 0.085 | 0.097 |
|  | (-0.040 - 0.215) | (-0.048 - 0.207) | (-0.040 - 0.217) | (-0.042 - 0.213) | (-0.033 - 0.227) |
| Age in years | -0.020*** | -0.020*** | -0.019*** | -0.020*** | -0.020*** |
|  | (-0.029 - -0.010) | (-0.029 - -0.010) | (-0.029 - -0.010) | (-0.029 - -0.010) | (-0.030 - -0.010) |
| Marital status: - married, not living together with spouse (Ref.: married and living together with spouse) | -0.049 | -0.051 | -0.047 | -0.048 | -0.044 |
|  | (-0.474 - 0.376) | (-0.476 - 0.374) | (-0.472 - 0.378) | (-0.473 - 0.377) | (-0.470 - 0.382) |
| - divorced | -0.039 | -0.042 | -0.038 | -0.041 | -0.094 |
|  | (-0.258 - 0.180) | (-0.260 - 0.176) | (-0.257 - 0.182) | (-0.260 - 0.177) | (-0.315 - 0.126) |
| - widowed | 0.070 | 0.095 | 0.071 | 0.072 | 0.047 |
|  | (-0.169 - 0.309) | (-0.145 - 0.336) | (-0.169 - 0.310) | (-0.167 - 0.312) | (-0.197 - 0.291) |
| - single | -0.225+ | -0.209+ | -0.209+ | -0.207+ | -0.248* |
|  | (-0.458 - 0.008) | (-0.441 - 0.023) | (-0.441 - 0.023) | (-0.440 - 0.027) | (-0.486 - -0.009) |
| Number of illnesses | -0.022 | -0.020 | -0.022 | -0.022 | -0.007 |
|  | (-0.057 - 0.013) | (-0.055 - 0.015) | (-0.057 - 0.013) | (-0.057 - 0.013) | (-0.043 - 0.029) |
| Mean monthly net equivalent income | 0.000 | 0.000 | 0.000 | 0.000 | 0.000 |
|  | (-0.000 - 0.000) | (-0.000 - 0.000) | (-0.000 - 0.000) | (-0.000 - 0.000) | (-0.000 - 0.000) |
| Occupational status: - retired (Ref.: employed) | 0.291** | 0.295** | 0.292** | 0.295** | 0.335** |
|  | (0.084 - 0.498) | (0.089 - 0.502) | (0.086 - 0.498) | (0.089 - 0.502) | (0.126 - 0.545) |
| - others | 0.238* | 0.234* | 0.238* | 0.238* | 0.269* |
|  | (0.015 - 0.460) | (0.011 - 0.457) | (0.015 - 0.460) | (0.015 - 0.460) | (0.042 - 0.497) |
| Help around house: yes (Ref.: no) | 0.004 |  |  |  |  |
|  | (-0.126 - 0.134) |  |  |  |  |
| Looking after someone: yes (Ref.: no) |  | -0.112 |  |  |  |
|  |  | (-0.274 - 0.050) |  |  |  |
| Nursing care services: yes (Ref.: no) |  |  | 0.009 |  |  |
|  |  |  | (-0.129 - 0.147) |  |  |
| Any other help: yes (Ref.: no) |  |  |  | -0.011 |  |
|  |  |  |  | (-0.141 - 0.119) |  |
| Time per week spent for informal care (in hours) |  |  |  |  | -0.004* |
|  |  |  |  |  | (-0.008 - -0.001) |
| Constant cut1 | -3.019*** | -3.155*** | -3.004*** | -3.033*** | -3.130*** |
|  | (-3.613 - -2.426) | (-3.771 - -2.540) | (-3.638 - -2.369) | (-3.631 - -2.436) | (-3.724 - -2.536) |
| Constant cut2 | -2.379*** | -2.519*** | -2.363*** | -2.393*** | -2.433*** |
|  | (-2.958 - -1.800) | (-3.119 - -1.918) | (-2.986 - -1.741) | (-2.976 - -1.811) | (-3.008 - -1.858) |
| Constant cut3 | -2.044*** | -2.182*** | -2.025*** | -2.058*** | -2.087*** |
|  | (-2.619 - -1.468) | (-2.779 - -1.586) | (-2.644 - -1.406) | (-2.637 - -1.480) | (-2.657 - -1.516) |
| Constant cut4 | -1.831*** | -1.969*** | -1.813*** | -1.845*** | -1.871*** |
|  | (-2.405 - -1.257) | (-2.564 - -1.375) | (-2.430 - -1.195) | (-2.422 - -1.269) | (-2.440 - -1.303) |
| Constant cut5 | -0.693* | -0.830** | -0.678* | -0.710* | -0.717* |
|  | (-1.262 - -0.125) | (-1.419 - -0.241) | (-1.290 - -0.066) | (-1.282 - -0.139) | (-1.280 - -0.154) |
|  |  |  |  |  |  |
| Observations | 1,246 | 1,247 | 1,248 | 1,246 | 1,209 |
| Pseudo R² | 0.009 | 0.009 | 0.008 | 0.008 | 0.011 |

Comments: Coefficients were reported (larger values correspond to "higher" outcomes). 95% confidence intervals in parentheses. *** p<0.001, ** p<0.01, * p<0.05, + p<0.10.
