# Supplementary material for: Association of informal caregiving with body mass index and frequency of sporting activities: evidence of a population-based study in Germany
Source: BMC Public Health. 2017 Sep 29;17:755. doi: 10.1186/s12889-017-4786-6 (PMC5622456; doi:10.1186/s12889-017-4786-6)
Supplement: Supplementary file 3 — Multiple ordered probit regression analyses with frequency of strenuous physical activities as dependent variable. (DOCX 15 kb) [file 12889_2017_4786_MOESM3_ESM.docx]

Additional file 3. Multiple ordered probit regression analyses with frequency of strenuous physical activities as dependent variable

|  | (1) | (2) | (3) | (4) | (5) |
| --- | --- | --- | --- | --- | --- |
| Independent variables | Dependent variable: Frequency of strenuous physical activities | | | | |
| Sex: female (Ref.: male) | -0.067 | -0.070 | -0.077 | -0.061 | -0.095 |
|  | (-0.190 - 0.057) | (-0.193 - 0.054) | (-0.201 - 0.048) | (-0.184 - 0.062) | (-0.221 - 0.030) |
| Age in years | -0.009+ | -0.009+ | -0.009* | -0.009+ | -0.010* |
|  | (-0.019 - 0.000) | (-0.019 - 0.000) | (-0.019 - -0.000) | (-0.018 - 0.000) | (-0.019 - -0.000) |
| Marital status: - married, not living together with spouse (Ref.: married and living together with spouse) | -0.348+ | -0.354+ | -0.357+ | -0.343+ | -0.351+ |
|  | (-0.756 - 0.059) | (-0.762 - 0.053) | (-0.764 - 0.051) | (-0.751 - 0.064) | (-0.759 - 0.057) |
| - divorced | -0.153 | -0.150 | -0.141 | -0.139 | -0.120 |
|  | (-0.362 - 0.057) | (-0.359 - 0.059) | (-0.351 - 0.070) | (-0.348 - 0.070) | (-0.332 - 0.091) |
| - widowed | 0.095 | 0.118 | 0.107 | 0.098 | 0.156 |
|  | (-0.133 - 0.323) | (-0.112 - 0.347) | (-0.122 - 0.335) | (-0.130 - 0.327) | (-0.077 - 0.389) |
| - single | -0.187 | -0.209+ | -0.205+ | -0.179 | -0.187 |
|  | (-0.415 - 0.040) | (-0.436 - 0.017) | (-0.432 - 0.021) | (-0.407 - 0.049) | (-0.420 - 0.046) |
| Number of illnesses | -0.025 | -0.025 | -0.026 | -0.028 | -0.022 |
|  | (-0.059 - 0.009) | (-0.059 - 0.009) | (-0.060 - 0.008) | (-0.062 - 0.006) | (-0.057 - 0.013) |
| Mean monthly net equivalent income | -0.000 | -0.000 | -0.000 | -0.000 | -0.000 |
|  | (-0.000 - 0.000) | (-0.000 - 0.000) | (-0.000 - 0.000) | (-0.000 - 0.000) | (-0.000 - 0.000) |
| Occupational status: - retired (Ref.: employed) | 0.126 | 0.120 | 0.115 | 0.122 | 0.106 |
|  | (-0.073 - 0.325) | (-0.078 - 0.319) | (-0.083 - 0.314) | (-0.077 - 0.321) | (-0.094 - 0.307) |
| - others | 0.136 | 0.130 | 0.130 | 0.128 | 0.105 |
|  | (-0.077 - 0.348) | (-0.083 - 0.342) | (-0.083 - 0.342) | (-0.085 - 0.340) | (-0.111 - 0.322) |
| Help around house: yes (Ref.: no) | 0.003 |  |  |  |  |
|  | (-0.123 - 0.129) |  |  |  |  |
| Looking after someone: yes (Ref.: no) |  | -0.120 |  |  |  |
|  |  | (-0.276 - 0.037) |  |  |  |
| Nursing care services: yes (Ref.: no) |  |  | -0.104 |  |  |
|  |  |  | (-0.237 - 0.030) |  |  |
| Any other help: yes (Ref.: no) |  |  |  | -0.117+ |  |
|  |  |  |  | (-0.242 - 0.008) |  |
| Time per week spent for informal care (in hours) |  |  |  |  | 0.003+ |
|  |  |  |  |  | (-0.001 - 0.006) |
| Constant cut1 | -2.291*** | -2.432*** | -2.487*** | -2.432*** | -2.351*** |
|  | (-2.856 - -1.726) | (-3.018 - -1.845) | (-3.095 - -1.879) | (-3.001 - -1.864) | (-2.912 - -1.791) |
| Constant cut2 | -1.524*** | -1.670*** | -1.725*** | -1.664*** | -1.559*** |
|  | (-2.080 - -0.967) | (-2.248 - -1.092) | (-2.325 - -1.125) | (-2.223 - -1.104) | (-2.110 - -1.008) |
| Constant cut3 | -1.217*** | -1.363*** | -1.419*** | -1.356*** | -1.253*** |
|  | (-1.771 - -0.663) | (-1.939 - -0.788) | (-2.017 - -0.821) | (-1.914 - -0.799) | (-1.802 - -0.705) |
| Constant cut4 | -0.911** | -1.058*** | -1.114*** | -1.053*** | -0.943*** |
|  | (-1.464 - -0.359) | (-1.632 - -0.483) | (-1.710 - -0.517) | (-1.608 - -0.497) | (-1.490 - -0.396) |
| Constant cut5 | 0.203 | 0.059 | 0.004 | 0.066 | 0.183 |
|  | (-0.349 - 0.756) | (-0.514 - 0.633) | (-0.591 - 0.599) | (-0.489 - 0.621) | (-0.363 - 0.730) |
|  |  |  |  |  |  |
| Observations | 1,251 | 1,252 | 1,253 | 1,251 | 1,214 |
| Pseudo R² | 0.004 | 0.004 | 0.005 | 0.005 | 0.004 |

Comments: Coefficients were reported (larger values correspond to "higher" outcomes). 95% confidence intervals in parentheses. *** p<0.001, ** p<0.01, * p<0.05, + p<0.10.
